# Clinical and Histological Long-Term Follow-Up of De Novo HBV-Infection after Liver Transplantation

**DOI:** 10.3390/medicina57080767

**Published:** 2021-07-28

**Authors:** Ramin Raul Ossami Saidy, Franziska Eurich, Maximilian Paul Postel, Eva Maria Dobrindt, Jasper Feldkamp, Selina Johanna Schaper, Johann Pratschke, Brigitta Globke, Dennis Eurich

**Affiliations:** Department of Surgery, Campus Virchow-Klinikum and Campus Charité Mitte, Charité-Universitätsmedizin Berlin, Augustenburger Platz 1, 13353 Berlin, Germany; raminraul.ossamisaidy@alumni.charite.de (R.R.O.S.); franziska.eurich@charite.de (F.E.); maximilian.postel@charite.de (M.P.P.); eva-maria.dobrindt@charite.de (E.M.D.); jasper.feldkamp@charite.de (J.F.); selina.schaper@charite.de (S.J.S.); johann.pratschke@charite.de (J.P.); brigitta.globke@charite.de (B.G.)

**Keywords:** viral hepatitis, liver transplantation, de novo hepatitis B infection, long-term follow-up

## Abstract

*Background and Objectives*: Development of hepatitis-B is considered a serious complication after liver transplantation. HBV de novo infection is a rather rare phenomenon, however it deserves attention in the era of donor organ shortage. The aim of the present analysis was to examine its course in liver transplant patients. *Materials and Methods*: Prevalence of de novo HBV-infections was extracted from our local transplant data base. Analysis focused on the moment of HBV-detection and on the long-term follow-up in terms of biochemical and histological changes over 30 years. *Results*: 46 patients were identified with the diagnosis of de novo hepatitis B. Median time from liver transplantation to diagnosis was 397 days (7–5505). 39 patients received antiviral therapy. No fibrosis progression could be detected, whereas the grade of inflammation significantly lessened from the moment of HBV detection to the end of histological follow-up over a median of 4344 days (range 123–9490). Patients with a poor virological control demonstrated a significantly poorer overall survival. *Conclusions*: De novo hepatitis B in liver transplant patients is a condition that can be controlled very well without significant fibrosis progression or graft loss if recognized on time within a regular transplant follow-up schedule.

## 1. Introduction

More than 300 million people are infected with the hepatitis B virus (HBV). The clinical course is categorized into acute HBV-associated liver failure, acute self-limited hepatitis B and chronic hepatitis B infection [1]. Chronic hepatitis B is hallmarked by constant viral replication with hepatitis B viremia and serological proof of HBV surface antigen (HbsAg) [2].

In patients with HBV-associated end stage liver disease (ESLD) liver transplantation (LT) is required, but reinfection may occur. The reinfection of the graft is believed to be endogenous, arising from an extrahepatic viral reservoir [3]. Continuous improvement of prophylaxis lowered the risk for HBV-reinfection from 90% to about 10%, still leaving the fear of graft and patient loss in cases of uncontrolled HBV-reinfection. Recent guidelines recommend prophylactic use of nucleos(t)ide analogues (NA) with higher barrier to resistance in combination with hepatitis B immunoglobulin (HBIG) in these patients [4].

De novo HBV-infection after LT however describes HBV-infection in a patient without prior exposition to the virus. Here, source of HBV infection may be transplantation of an HBcAb-positive (HBcAb+) graft without adequate NA-based prophylaxis; the virus may reactivate under immunosuppression transmitted from the graft hepatocytes, leading to the-mostly chronic-inflammation of the whole graft. Nevertheless, these extended criteria grafts may show an excellent function and provide a non-inferior patient survival, if HBV-reactivation is prevented by the use of NA [5]. Last, HBV infections in liver transplant recipients may also be transmitted in a graft unrelated fashion, i.e., by contact with contaminated body fluids, as sexually transmitted disease by blood transfusions or via other less common routes. Disregarding the mode of transmission, de novo HBV-infection is a feared complication that may progress to fulminant icteric hepatitis, and acute liver failure, especially under immunosuppression, as development of specific antibodies may be impaired [6].

Data on liver transplant patients with de novo HBV infection is scarce. Therefore, we present long-term histological, laboratory and clinical follow up of our cohort in order to close this gap.

## 2. Materials and Methods

Between 1988 and 2017, 3014 LTs have been performed in 2686 patients at the Charité-Universitätsmedizin Berlin, Campus Virchow-Klinikum, for the treatment of various liver diseases. A retrospective analysis was conducted and patients with de novo HBV infection were identified as those who underwent LT for other reasons than HBV-associated ESLDs and without record or indication of prior HBV-infection in laboratory testings but who were tested positive for HbsAg/HBV-DNA at least once after transplantation. HbsAg was measured by an immunoassay for the qualitative determination of hepatitis B surface antigen (Roche, Basel, Switzerland) and HBV-DNA was assessed by quantitative DNA-polymerase-chain-reaction. Thus, *n* = 46 patients that were successfully transplanted were identified.

*n* = 14 of this group had received an HBcAb+ graft. The 14 patients belonged to the collective of 78 successfully transplanted patients with HBcAb+ LT and without prior known HBV-infection.

**Figure 1:**
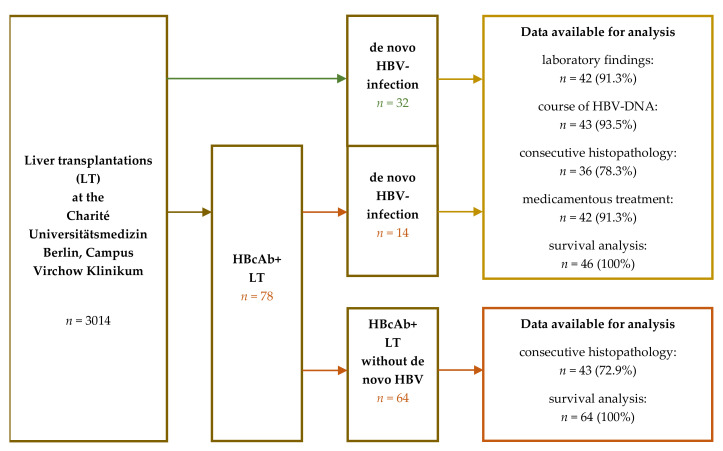
Patient collectives. From 1988 to 2017, *n* = 2686 patients underwent 3014 liver transplantations (LT) for various liver diseases. Among these, *n* = 46 patients that were successfully transplanted were reported with hepatitis B-Virus (HBV)-infection after LT without prior known infection. *n* = 14 of this group had received an hepatitis B core antibody (HBcAb)+ graft forming a collective of *n* = 78 successfully transplanted patients with HBcAb+ LT and without prior known HBV-infection. For analysis, *n* = 4 patients had left care of our outpatient clinic and follow-up was scarce. Consecutive histopathological control biopsies were available in *n* = 36 of patients with new HBV-infection. For comparison, patients with HBcAB+ LT without de novo infection were installed as a “control group”.

Consecutive histopathological control biopsies were available in *n* = 36 of patients with new HBV-infection. For comparison, patients with HBcAb+ LT without de novo infection were installed as a “control group”. See also Figure 1.

Diagnostics for HBV-infection were initiated due to increased levels of transaminases (TA), elevated bilirubine, clinical signs of infection and for routine check.

All patients were followed up at our outpatient department with regular visits for laboratory tests at intervals ranging from twice a week to once in 12 weeks in a time-dependent manner after LT. By routine, liver tissue samples were performed as percutaneous ultrasound-guided biopsies at 1, 3, 5, 7, 10, and so on years for an indefinite duration and on indication. Follow-up protocol was consistent for all patients in our transplant outpatient clinic.

Levels of TAs from laboratory tests were assessed and categorized into normal and elevated according to threshold parameters provided by the hospital’s laboratory. Elevation of either aspartate aminotransferase or alanine aminotransferase or both were considered “elevated”.

Liver biopsies performed as part of post-transplant care were evaluated by experienced pathologists. The assessment of inflammation grade and fibrosis stage was performed using the classification proposed by Desmet and Scheuer [7]. Grades of inflammation were classified as follows: 0—no inflammation; 1—minimal; 2—mild; 3—moderate; and 4—severe. Fibrosis was staged on a scale of 0–4: 0, absent; 1—mild portal fibrosis; 2—moderate with few incomplete portal septa; 3—numerous portal septa without architectural disturbances; and 4—cirrhosis. Steatosis was assessed as follows: 1—<30%, 2—<60% and 3—>60%.

Descriptive analysis was performed to calculate the median age of patients at transplantation, HBV infection, and days of follow-up. Continuous variables were assessed using the t-test or Mann-Whitney U-test (in case of skewed data). The Wilcoxon signed-rank test was used to detect differences in paired categorical variables. Categorical variables were compared using cross tables. Kaplan–Meier analysis with Log-rank-test was performed to compare and illustrate survival differences. A *p*-value (two-sided) of <0.05 was considered to be statistically significant. Statistical analysis was conducted using the SPSS software version 26 (IBM, Endicott, New York, USA).

The study was performed retrospectively according to the Professional Code of the German Medical Association (article B.III.§15) based on the World Medical Association’s Declaration of Helsinki and was approved by the local Ethics Committee of Charité Universitätsmedizin Berlin (protocol code EA1/035/21; date of approval 3 November 2020).

## 3. Results

Out of 3014 liver transplantations in 2686 patients, *n* = 32 patients (1.2%) demonstrated a de novo HBV infection after LT without obvious source of infection. Additionally, *n* = 78 (2.9%) of patients received a HBcAb-positive graft without having undergone HBV-infection prior to LT. In this subset, *n* = 14 (17.9%) patients were recorded with HBV infection in terms of reactivation from the anti-HBc-positive graft. This constitutes a prevalence of 1.7% (*n* = 46) of de novo Hepatitis B-infection in the entire LT cohort of 2686 recipients (see Table 1 and Figure 1). In this cohort, the most frequent indication for transplantation was non-viral cirrhosis (*n* = 24/52.2%) or underlying autoimmune disease (*n* = 11/23.9%). All patients were routinely tested negative for HbsAg/HBV-DNA at the time of transplantation. Median time from LT to the diagnosis of de novo HBV infection was 397 days (7–5505), whereas the median duration of infection (time span from first diagnosis to death or end of follow-up) was 2550 days (44–8760).

At the end of the observation period, a liver associated cause of death was reported in *n* = 5 (10.9%) out of *n* = 19 fatal cases (41.3%). Other causes of death included cancer (*n* = 6/13%) sepsis (*n* = 3/6.5%), alcoholism (*n* = 2/4.3%) and cardiovascular disease (*n* = 3/6.5%).

One patient had died very quickly after initial diagnosis of HBV-infection due to metastasized HCC, three patients were lost to follow-up and information of de novo HBV was received via consultations of treating physician. However, in these cases, treatment regimen remained unclear.

After the diagnosis of de novo HBV infection, in *n* = 42 (91.3%), pharmaceutical treatment was evaluated. Laboratory proof of HBV-DNA/HBsAG was seen as indication for treatment. *n* = 25 (54.3%) were treated with oral monotherapy of NAs, with *n* = 14 (30.4%) receiving a high-genetic barrier NA, either tenofovir (TDF) or entecavir (ETV). Combination therapy of NAs was administered in *n* = 14 (30.4%) and two patients (4.3%) needed re-LT, one because of fulminant HBV-Infection, one because of recurring primary sclerosing cholangitis (PSC). In one case, no treatment was initiated due to patient’s wish.

Status of HBV-infection at the end of follow-up was available in *n* = 43 (93.5%) patients; in 32 (69.6%) cases, HBV-DNA was undetectable with PCR, indicating successful therapy, in eleven (23.9%) patients the infection persisted. HBsAg seroconversion was observed in 13 (28.3%) patients in long-term follow-up while *n* = 30 (65.2%) remained anti-Hbs negative at the time of the last control (see Table 2).

**Figure 2:**
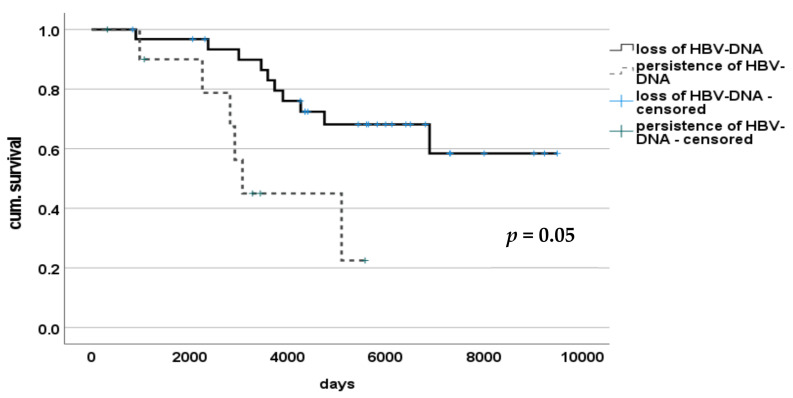
Survival of patients with or without de novo HBV-infection persistence after LT.

Kaplan-Meier analysis of patients with de novo HBV-infection after liver transplantation revealed significant longer survival of patients with loss of HBV-DNA in serum during treatment (*n* = 32) compared to patients with persistence of HBV-DNA in peripheral blood (*n* = 12), (*p* = 0.005). Median survival was 5090 days (834–9490) and 2917 days (317–5573) respectively (Figure 2). In patients with loss of HBV-DNA, only one (10%) died of a liver associated death, while in the group with persistent HBV-infection, out of seven patients that died four deaths (57.1%) were registered as liver-associated. This difference reached statistical significance (*p* = 0.036).

In *n* = 42 (91.3%) patients analysis of consecutive laboratory findings starting at diagnosis of de novo HBV-infection up to the last follow-up was available. Aminotransferases were elevated at diagnosis in *n* = 25 (54.3%) and remained so in *n* = 15 (32.6%) at the end of follow-up. A statistically significant reduction of TAs between these time points (*p* = 0.025) was observed.

Biopsy results for histopathological assessment were available in *n* = 37 (80.4%) patients at the beginning of HBV infection and in *n* = 36 (78.3%) at the end of follow-up. Absence of inflammation was seen in one (2.2%) patient, minimal inflammation in 10 (21.7%) biopsies, in *n* = 21 (45.7%), inflammation grade was 2 and in three (8.7%) inflammatory reaction was classified as moderate and one case was scored with grade 4. In follow-up biopsies, inflammation grade 0 was found in *n* = 11 (23.9%), minimal inflammation in *n* = 13 (28.3%), mild in *n* = 11 (23.9), and one (2.1%) patient showed inflammatory grade 4. Analysis of paired variables showed a significant reduction (*p* = 0.001) of inflammatory grades during the clinical course.

In four patients (8.7%) no histological stage of fibrosis was found at initial diagnosis, 19 (41.3%) patients were diagnosed with grade 1, nine (19.6%) patients showed stage 2 and five (10.9%) stage 3 of fibrosis. Observation controls revealed stage 0 in eight cases (17.4%), portal fibrosis in *n* = 16 (34.8%) biopsies, stage 2 in seven (15.2%) patients and in three (6.5%) stage 3 was diagnosed. In two patients (4.5%), an apparent progression of fibrosis into liver cirrhosis (stage 4) was stated. Wilcoxon test revealed no statistically significant changes regarding fibrosis stages between the beginning of HBV-infection and last biopsy result (*p* = 0.41).

**Figure 3:**
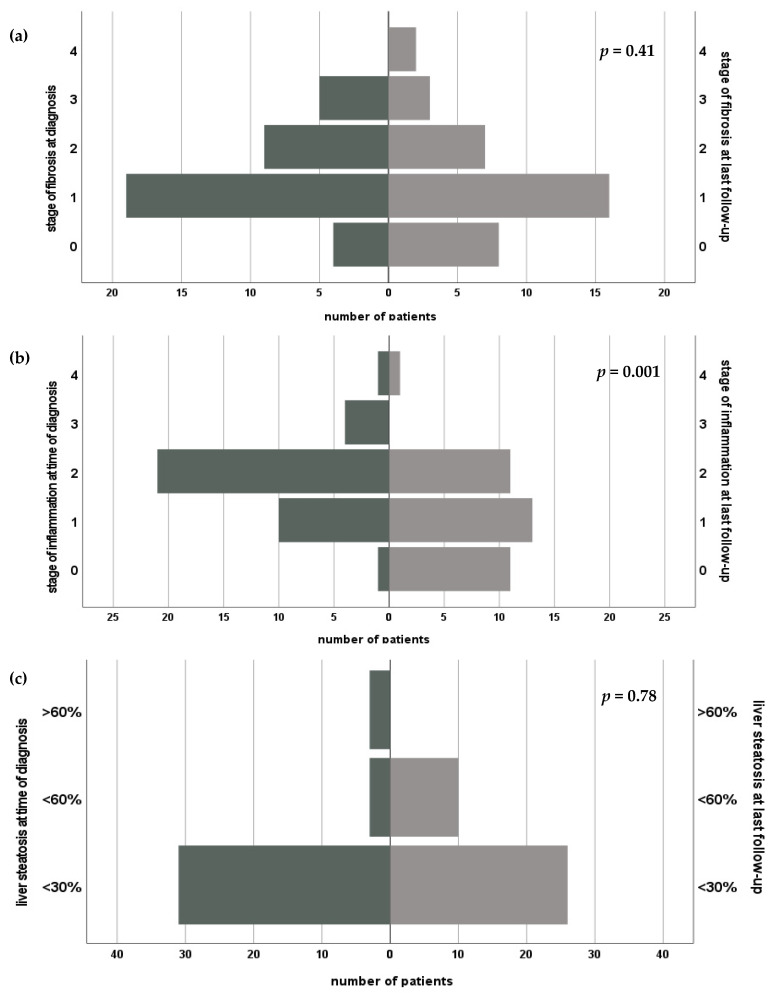
Course of fibrosis (**a**), inflammation grade (**b**) and steatosis hepatis (**c**) after diagnosis of de novo HBV after liver transplantation.

Liver steatosis <30% at diagnosis of de novo HBV-infection was seen in *n* = 31 (67.4%) and in *n* = 26 (56.5%) at the time of the last follow up. Three (6.5%) patients showed steatosis >60% in the beginning, but none at the last follow-up. However, statistical analysis showed no significant change over time (*p* = 0.78). Results of the histological analysis are displayed in Figure 3.

**Figure 4:**
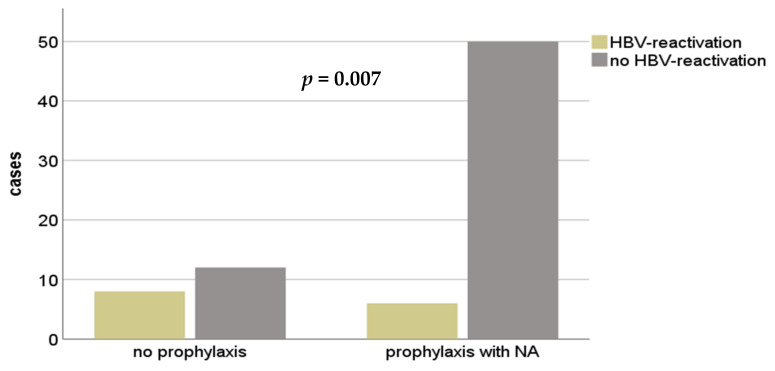
Recurrent HBV-infection in patients after HBcAb+ LT with or without a prophylactic therapy. NA—nucleos(t)ide analog; HBV—hepatitis B Virus; HBcAb—hepatitis B core antibody.

In a subgroup analysis of *n* = 78 patients, who received a successful liver transplantation with a HBcAb+ graft, 14 (17.9%) patients were identified with de novo HBV-infection in terms of a HBV reactivation. 56 (71.8%) recipients received prophylaxis (e.g., lamivudin (LAM) or adenofovir (ADV) monotherapy) after LT and *n* = 20 (25.6%) did not. In two patients, no documentation was available. De novo HBV-Infection occurred in *n* = 8 (40.0%) without prophylaxis and in six (10.7%) patients despite NA-therapy. This difference proved to be statistically significant (*p* = 0.007), (see Figure 4). After HBV-reactivation all patients received specific antiviral therapy; in ten (71.4%) NA-monotherapy and in four (28.6%) combination of NAs was administered. Here, TDF was used predominantly for monotherapy (*n* = 8).

At the end of follow-up, HBV-DNA was undetectable in 10 patients (71.4%), whereas persistent viremia was noted in four (28.6%) cases.

In this subgroup, aminotransferases at time of diagnosis of de novo HBV-infection were increased in nine (64.3%) patients, but only one (7.1%) patient showed increased levels at last follow-up. This transformation proved to be statistical significant (*p* = 0.005).

For histopathological course, *n* = 10 (71.4%) consecutive biopsies were available. No statistical significant differences regarding change of stage of fibrosis (*p* = 0.16) or liver steatosis (*p* = 0.32) was found. Decrease of inflammation grade over time showed statistical significance (*p* = 0.014).

Kaplan-Meier analysis showed no significant difference in survival between the 14 patients undergoing antiviral therapy due to de novo HBV infection and 64 patients without HBV-infection after HBcAb+ LT (*p* = 0.063). Median survival time was 3583 days (834–5820) and 2133 days (150–6270) respectively.

For comparison of the histopathological course of patients with de novo HBV infection, *n* = 36 patients were matched with the collective of *n* = 59 patients receiving a HbcAb+ liver transplant as a “control group” due to homogenous profiles such as age and indication to LT.

Thus, 36 consecutives biopsies of patients with new onset of HBV-infection after LT (Group HBV) were compared with routine control biopsies of 43 patients after HBcAB+ LT (controls).

Median histology time span of observation was significantly longer in patients with de novo HBV-infection than in control group (*p* < 0.001) (2362 (106–8045) vs. 1825 (184–3654) days; *p* < 0.001).

There was no significant difference between groups regarding fibrosis stages at the first (*p* = 0.27) or the last biopsy (*p* = 0.38). Similarly, the extent of liver steatosis did not differ between groups in first (*p* = 0.87) or last biopsy (*p* = 0.15). Inflammatory extend showed significantly higher levels in patients with de novo HBV-infection at time of diagnosis compared to routine biopsies of the control group (*p* < 0.001). However, comparison at the time of the last follow-up, showed no difference (*p* = 0.116), indicating a return of inflammation levels to the baseline of LT patients.

**Figure 5:**
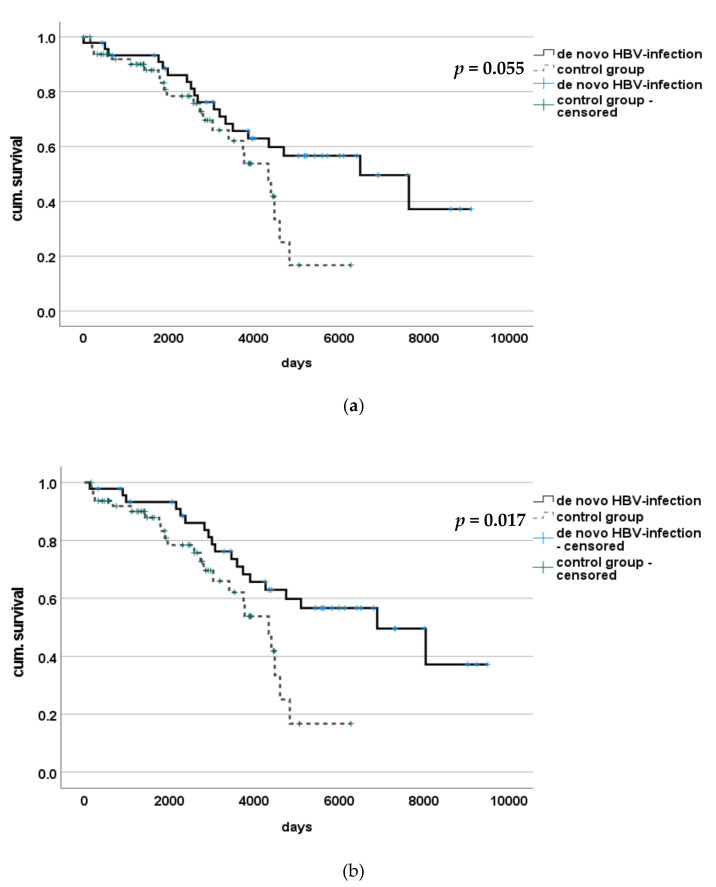
Survival of patients with or without de novo HBV-infection after LT. (**a**) Statistical significant difference in overall survival after liver transplantation was found between patients with de novo HBV-infection and those without. (**b**) After adjusting to median time of discontinuation (397 days) to better evaluate course after de novo HBV-infection survival did not show any significant difference.

Kaplan-Meier analysis showed a significant difference in survival between patients with de novo HBV-infection and those without, with estimated median survival for the HBV group of 4304 (123–9490) days and 2133 (150–6270) days in the control group (*p* = 0.017). After adjustment of time of survival to median time to infection (397 days), survival between these two groups no longer differed with statistical significance with 3907 (0–9093) days and 2133 (150–6270) days, respectively (*p* = 0.055) (see Figure 5).

No significance was found when Group HBV was split in a time-dependent manner in regard to available therapeutical options at time of diagnosis (high-genetic-barrier NA); there was no longer survival for those patients with de novo HBV-infection after 2005 compared to those with diagnosis between 1988–2005 (*p* = 0.68).

## 4. Discussion

The most common route of hepatitis B transmission in patients after LT is through the graft itself [8]. Virus reactivation can be observed frequently under immunosuppressive therapy after transplantation. Postoperative presence of HBV DNA and HbsAg in the recipients’ blood is a standard indicator of HBV infection. Despite the relatively low prevalence of 1.7% of de novo HBV infection in our LT cohort, it still constitutes an important finding. In contrast to our results, other studies have reported higher prevalence of up to 6.5% of de novo HBV infection, which may be explained by epidemiological differences across countries [9,10]. Furthermore, a remarkable heterogenity exists regarding the definition of de novo HBV infection, thus possibly explaining the differences. While large clinical data for reactivation of HBV after LT from an antiHBc-positive donors exists, data on de novo hepatitis is scarce [11]. A recent article found a prevalence of 10.7% of de novo HBV after LT in a collective of 159 patients; here mortality was higher in affected patients [12].

In the present study of this unique population, the group of de novo HBV infection comprised patients with HBV-reactivation from an antiHBc-positive donor and patients without obvious source of infection in order to assess histopathological changes in the graft and deliver robust long-term information based on the needle biopsy.

Chronic hepatitis B impairs hepatocyte function after years of active virus replication, leading to progression of fibrosis. Moreover, rapid organ failure can be observed due to hepatitis infection in combination with immunosuppressive therapy in patients after LT. Fibrosing cholestatic hepatitis (FCH) is hallmarked by a fast progression of hepatocellular injury, severe cholestasis, and periportal and cellular fibrosis in histopathological tests [6]. However, recently published articles stated that no fibrosis progression occurred in patients with HBV reinfection who were transplanted for HBV-associated ESLD [13,14]. These findings are in accordance with our data on the de novo HBV infection after LT. There is little report of histological courses of de novo HBV-infection after LT and to our knowledge, we are able to provide the largest cohort with consistent follow-up for this occurrence. We did not observe any significant progress in steatosis or inflammation, with even a significant decrease in inflammatory signs after initiation of therapy. Hence, if HBV infection is diagnosed early, and treatment is immediately initiated, serious damage to the graft can be prevented as other-reports have stated earlier [15,16]. For this desirable outcome, the need for thorough, life-long follow-up after LT becomes evident.

In our study, we performed continuous laboratory tests post-LT, including HBV serology and liver biopsy, to detect and quantify early HBV-associated graft damage. De novo HBV infection can be diagnosed by elevated liver enzyme levels even in patients who are transplanted for reasons other than HBV. However, liver biopsy still remains the gold standard for not just diagnosing but also ruling out acute rejection and quantifying the grade of inflammation and stage of fibrosis. Oral drug treatment with NAs is based on the inhibition of HBV replication and shows a success rate of up to 94% in non-LT patients [17]. In 32 out of 46 patients (69.6%), HBV control was successfully obtained with LAM, ADV), and/or TDF used as mono- or combination therapy.

Hepatitis B treatment has significantly improved after the introduction of high genetic barrier NAs, such as ETV and TDF, in 2006 and 2008, respectively. Although, currently, TDF and ETV provide the first-line therapy for chronic HBV infection, these were not available in the early 1990s, thereby leading to low treatment rates. Moreover, TDF reduces the viral load even when treatment with LAM fails due to rapid development of resistant species. Consistent with this finding, previous studies showed a regression of fibrosis and cirrhosis under TDF treatment in patients with chronic HBV-infection without liver transplantation [18]. This is confirmed in our histopathological controls, where no significant progression of fibrosis was observed and the majority of patients only demonstrated mild stages of fibrosis. Thus, with the administration of NAs, viral control and hence HBV treatment was possibly more effective during this data collection period (30 years).

Finally, in our cohort, the overall survival depended on the success of viral control. Patients who were HBVAg- or DNA-negative at the end of the observation had significantly better survival rates. Survival was reduced significantly, if HBV replication control was not sufficient, thus highlighting the importance of early diagnosis and treatment initiation. We also analyzed survival of patients with new onset of HBV-infection with a control group of patients receiving an HBcAb+ organ. When adjusted to time of infection, overall patient survival did not differ, indicating a diminishing impact of HBV-infection on course after LT in the era of replication control.

A recent study showed that HBV-naive children who received an HBcAb+ organ had minimal hepatitis B associated complications due to vaccination before and prophylaxis after the transplantation. In an era of organ shortage, increasing the acceptance of HBcAb+ organs by ensuring postoperative Hepatitis B prophylaxis can help to use the existing donor pool to full capacity [19]. Our histopathological findings, that showed no significant progression of fibrosis, further implicate a favorable long-term function.

HBV prevention should be administered in patients after LT for HBV-associated ESLDs and anti-HBc+ donor status. We observed significantly lower HBV reactivation from an antiHBc-positive graft when prophylaxis was administered compared to a regimen with our prophylaxis. As a prophylactic regimen is mandatory in this subgroup, low-genetic barrier NAs (e.g., lamivudine) are sufficient in most cases [3]. In case of HBV-infection, a high genetic barrier NA (e.g., TDF) should be used for replication control.

Under a common immunosuppressive regimen consisting of corticosteroids, calcineurin inhibitors, mycophenolat mofetil and/or m- TOR inhibitors in different combinations, the immune system is compromised to a degree that sufficient HbsAb-titers are not achieved in most cases [20]. Higher response rates of up to 80% were observed with a new generation of hepatitis B vaccines when adjuvants, such as 3-deacylated monophosphoryl lipid A (MPL) and Quillaja saponaria (QS21) were used [21,22]. Improvements in active vaccination that aim for a natural and long-lasting immunity are a promising outlook. However, further research to develop new hepatitis B vaccines and prospective studies for testing their efficiency are needed [20]. Therapy of de novo infections in these patients however is still feasible and we did not observe a worsened outcome in survival time or histopathological features of ongoing liver tissue damage.

Of note, in cases of transplantation of a HBcAb+ graft, de novo HBV-infection could be regarded as “transmission” rather than a new onset infection, as the initial infection of the liver has occurred in the donor. As an infection occurs in a naïve recipient, we hypothesize a course equivalent to other de novo infections, where transmission remains unclear or occult HBV infection (OBI) is assumed.

Limitations of the study are of course the retrospective character of data analysis and the limited number of patients. This is why two types of cohorts with HBV infection in patients after LT for ESLD not related to HBV were included in the analysis, demonstrating lack of fibrosis progression during a long term follow-up period with a moderate number of drop-outs due to loss to follow-up or death. The survival results may be biased by the choice of recipients of HBcAB+ grafts, because of a significantly different distribution of the diagnosis leading to LT, as patients with HCC where more likely to be chosen to receive an extended criteria graft. Still, its profile was homogeneous and feasible for thorough analysis.

## 5. Conclusions

In this study, we showed the need for consistent and ongoing medical aftercare of patients with de novo HBV-infections in a well-observed population. De novo HBV-infection, if recognized on time, and if treated appropriately, does not have a negative impact on development of fibrosis and on overall survival after LT. Thus, impact of de novo infection has changed drastically with the feasibility of highly effective antiviral therapies and, if monitored closely, the former fear of HBV-infection in these patients may no longer be relevant in the future.

## Figures and Tables

**Table 1:** Patient collectives and general characteristics.

	De Novo HBV-Infection after LT	HbcAb+ LT without De Novo HBV-Infection	*p*
	*n* = 46	*n* = 64	
Sex			0.401
female	*n* = 17 (37.0%)	*n* = 21 (33.8%)	
male	*n* = 29 (63.0%)	*n* = 43 (66.2%)	
Mean age at LT (std)	49.26 years (11.5)	54.43 years (12.8)	0.031
LT Indication			0.005
cirrhosis	*n* = 24 (52.2%)	*n* = 29 (45.3%)	
HCC	*n* = 3 (6.5%)	*n* = 15 (23.4%)	
HCV	*n* = 3 (6.5%)0	*n* = 5 (7.8%)	
autoimmune	*n* = 11 (23.9%)	*n* = 4 (6.3%)	
other	*n* = 4 (8.7%)	*n* = 11 (17.2%)	
Backbone immune suppression			
CNI	*n* = 40 (87.0%)	*n* = 63 (98.4%)	
MMF	*n* = 3 (6.5%)	*n* = 1 (0.6%)	0.042
Cortison	*n* = 3 (6.5%)	*n* = 0 (0%)	0.06
Combination therapy	30 (65.2%)	31 (48.4%)	
Reasons for Diagnosis of HBV			n.a
elevetaed TAs	*n* = 25 (54.3%)		
elevated bilirubine/icterus	*n* = 2 (4.3%)		
clinical signs of infection	*n* = 9 (19.6%)		
routine check	*n* = 2 (4.3%)		
Median Observation Period min-max)	4344 days (123–9490)	2133 days (150–6270)	<0.001
Interquartile range Q1/Q3	2708.5/6191.5 days	1140/3499.25 days	
Median histological observation (min-max)	2362 days (106–8045)	1825 (184–3654)	<0.001
Status in last follow-up			0.69
alive	*n* = 27 (58.7%)	*n* = 41 (64.1%)	
deceased	*n* = 19 (41.3%)	*n* = 23 (35.9%)	
HBcAb-Status of liver transplant			n.a.
HBcAb+	*n* = 14 (30.4%)	*n* = 64 (100%)	
HBcAb−	*n* = 32 (69.6%)	*n* = 0 (0%)	
HbsAG/HBV-DNA in last follow-up			n.a.
HbsAG/HBV-DNA+	*n* = 32 (69.6%)	*n* = 0 (0%)	
HbsAG/HBV-DNA−	*n* = 11 (23.9.6%)	*n* = 64 (100%)	
no information	*n* = 3 (6.5%)		

CNI—calcineurin inhibitor; HCC—hepatocellular carcinoma; HCV—Hepatitis C Virus; HBV—Hepatitis B Virus; LT—liver transplantation; MMF—mycophenolate-mofetile; n.a—not applicable; TA—transaminase.

**Table 2:** Parameters at time of diagnosis/treatment initiation of de novo HBV-infection after LT.

		De Novo HBV-Infection after LT *n* = 46
Diagnosis of HBV-infection		
HBsAg-positive		*n* = 46 (100%)
HBV-DNA available		*n* = 29 (63.0%)
Median laboratory parameters at diagnosis of de novo HBV-infection (min-max)		
	norm/threshold	
HBV-DNA	<100 cop/mL	7,056,000 (13,460–477,000,000)
ALT	<41 U/l	32 (8–256)
AST	<50 U/l	34 (11–402)
bilirubine	<1.2 mg/dl	0.7 (0.2–9.5)
platelets	150–370/nl	162.5 (96–1127)
INR	0.9–1.25 s	1.1 (0.88–1.6)
pharmacological treatment		
NA-monotherapy		*n* = 27 (58.7%)
TDF/ETV		*n* = 14 (30.4%)
ADV/LAM		*n* = 13 (28.3%)
NA-combination therapy		*n* = 14 (30.4%)
NA + NA		*n* = 13 (28.3%)
NA + HBIg		*n* = 1 (2.2%)
Re-LT		*n* = 2 (4.3%)
Treatment success		
HbsAg/HBV-DNA+		*n* = 32 (69.6%)
HbsAg/HBV-DNA−		*n* = 11 (23.9.6%)
HBsAg seroconversion		*n* = 13 (28.3%)

ALT—alanine aminotransferase; AST—aspartate aminotransferase; INR—international normalized ratio of Quick; TDF—tenofovir; ETV—entecavir; ADV—adenofovi; LAM—lamivudine; HBIg—Hepatitis B Immunoglubuline.

## Data Availability

The data presented in this study are available on request from the corresponding author. The data are not publicly available due to specifications of the local Ethics Committee and the institution’s data policy.

## Abbreviations

ADF	adenofovir
AIH	autoimmune hepatitis
ALD	alcoholic liver disease
ALF	acute liver failure
ALT	alanine aminotransferase
Anti-HBc	hepatitis B core antibody
Anti-HBe	hepatitis B e antibody
Anti-HBs	hepatitis B surface antibody
AST	aspartate aminotransferase
ETV	entecavir
ESLD	end stage liver disease
HBV	hepatitis B virus
HBcAb	hepatitis B core antibody
HBeAg	hepatitis B e antigen
HBIG	hepatitis B immunoglobulin
HBsAg	hepatitis B surface antigen
HCC	hepatocellular carcinoma
HCV	hepatitis C virus
IFN	interferone
ITBL	ischemic type biliary lesions
LT	liver transplantation
LAM	lamivudine
NA	nucleos(t)ide analog
NASH	nonalcoholic steatohepatitis
PBC	primary biliary cholangitis
PSC	primary sclerosing cholangitis
TA	transaminase
TDF	tenofovir
